# Customised fragments libraries for protein structure prediction based on structural class annotations

**DOI:** 10.1186/s12859-015-0576-2

**Published:** 2015-04-29

**Authors:** Jad Abbass, Jean-Christophe Nebel

**Affiliations:** Faculty of Science, Engineering and Computing, Kingston University, London, KT1 2EE UK

**Keywords:** Ab initio fragment-based protein structure prediction, Rosetta, Protein structural class, CATH, SCOP

## Abstract

**Background:**

Since experimental techniques are time and cost consuming, in silico protein structure prediction is essential to produce conformations of protein targets. When homologous structures are not available, fragment-based protein structure prediction has become the approach of choice. However, it still has many issues including poor performance when targets’ lengths are above 100 residues, excessive running times and sub-optimal energy functions. Taking advantage of the reliable performance of structural class prediction software, we propose to address some of the limitations of fragment-based methods by integrating structural constraints in their fragment selection process.

**Results:**

Using Rosetta, a state-of-the-art fragment-based protein structure prediction package, we evaluated our proposed pipeline on 70 former CASP targets containing up to 150 amino acids. Using either CATH or SCOP-based structural class annotations, enhancement of structure prediction performance is highly significant in terms of both GDT_TS (at least +2.6, p-values < 0.0005) and RMSD (−0.4, p-values < 0.005). Although CATH and SCOP classifications are different, they perform similarly. Moreover, proteins from all structural classes benefit from the proposed methodology. Further analysis also shows that methods relying on class-based fragments produce conformations which are more relevant to user and converge quicker towards the best model as estimated by GDT_TS (up to 10% in average). This substantiates our hypothesis that usage of structurally relevant templates conducts to not only reducing the size of the conformation space to be explored, but also focusing on a more relevant area.

**Conclusions:**

Since our methodology produces models the quality of which is up to 7% higher in average than those generated by a standard fragment-based predictor, we believe it should be considered before conducting any fragment-based protein structure prediction. Despite such progress, ab initio prediction remains a challenging task, especially for proteins of average and large sizes. Apart from improving search strategies and energy functions, integration of additional constraints seems a promising route, especially if they can be accurately predicted from sequence alone.

**Electronic supplementary material:**

The online version of this article (doi:10.1186/s12859-015-0576-2) contains supplementary material, which is available to authorized users.

## Background

Although the first protein structure was determined 56 years ago [1], experimental techniques are still time and cost consuming. Consequently, computational techniques are essential to produce conformations of protein targets. While excellent results can be produced in silico when homologous structures are available, despite advancements in the field of Bioinformatics, structure predictions remain far from being accurate and reliable when attempting to identify a protein’s native conformation from its sequence alone [2].

Ab initio methods (also known as de novo, template-free, or physics-based modelling) mimic Anfinsen’s thermodynamic principle by seeking the lowest possible energy conformation that a sequence can adopt [3]. Initially, physics-based methods were proposed, sampling the conformation space until reaching that minimal energy. Although successful predictions have been achieved using Monte Carlo methods and molecular dynamics simulations [4-6], their extensive computational requirements have limited their application to small proteins. Usage of approximations and heuristics has been a strategy to reduced computational costs; however this has led to the production of less accurate models. As a result, application of those approaches has been mainly limited to the study of the folding pathway of small proteins rather than prediction of final conformations [7]. To deal with those limitations, fragment-based methods with fast search techniques such as Monte Carlo simulations have been introduced to provide ‘coarse-grained’ ab initio predictions [8]. Evaluation in community-wide competitions has shown that fragment-based predictions perform well when dealing with short proteins [9]. As a consequence they have become the methods of choice when ab initio prediction is required. However, current approaches still have many limitations. We propose to address some of them by integrating structural constraints in their fragment selection process.

After a review of fragment-based protein structure prediction approaches and protein structure classifications, we propose the usage of structural classes to constrain standard fragment-based methods in order to reduce the size of conformation space they need to explore.

### Fragment-based protein structure prediction

Motivated by the fact there is a strong correlation between sequence and structure at the local level [10], fragment-based protein structure prediction methods were first proposed in 1994 by Bowie and Eisenberg [11]. They rely on the concatenation of short rigid fragments excised from actual protein structures to construct putative protein models. Since conformation space is explored at a fragment level, the entropy of the conformational search is reduced dramatically compared to standard ab-initio approaches. Still, unlike homology and threading modelling, fragment-based predictors are able to handle template-free modelling (FM) targets.

In order to eliminate the ‘discrete’ nature of the process of associating the best sub-structures to given sub-sequences, first, continuous overlapping fragments along the sequence are used, second, weighted knowledge-based energy functions are applied to measure the fitness of fragments using non-local interactions, and third, all-atom refinement is conducted [12]. Such procedure aims at emulating the actual protein folding mechanism which is believed to follow a ‘local-to-global/divide-and-conquer’ process which would explain the high speed of the folding process observed in nature [2,13,14]. Regarding the choice of fragment length, several studies concluded that their optimal size should be around 10 amino acids [15,16]. Moreover, it was shown that at least a set of 100 fragments should be explored for each position to produce native-like conformations [16].

According to performance [17] evaluated by the Critical Assessment of protein Structure Prediction (CASP) [18] - the community-wide biennial event which aims at objective evaluation of protein structure predictors -, FRAGFOLD can be considered as the first successful attempt in long fragment assembly protein structure prediction [19]. Moreover, since its initial participation in 1996, it has been continuously updated and remains an important CASP contributor [9]. FRAGFOLD’s main contribution has been the usage of two types of fragments: supersecondary structural motifs (variable length of 9 to 31 residues) which have been shown to be parts of the polypeptides that form early but remain stable during the folding process [20,21], and miscellaneous fragments extracted from high-resolution proteins (fixed length of 9-mers) [22-24].

Studies highlighting local sequence-structure relationships [25] suggested that methods built on Bowie and Eisenberg’s principles should only consider short fragments. As a result, Rosetta, a fully ab initio protein structure prediction suite, offered to generate conformations from assemblies of short fragments (3-mers and 9-mers) excised from high resolution protein structures [26]. Using the target’s sequence, for each position, the best 9-mers and 3-mers are selected. This is performed not only using the sequence profile, but also by considering secondary structure (SS) prediction information generated from several sources as well as Ramachandran map probabilities. Then, the process of building conformations is conducted using two levels of search and refinement: coarse and fine-grained associated with their respective energy functions. In the first level, low-resolution conformations are generated by representing the chain by heavy atoms of the backbone besides a single centroid for the side chains, whereas in the second one, all atoms are modelled. In addition to keeping the fragments rigid during the simulation as most methods do, Rosetta maintains bond angles and length at some ideal values to reduce the search space. Accordingly, the sole degrees of freedom in the coarse-grained search are the backbone torsion angles, whereas, side chains’ are only taken into account in the fine-grained stage [12]. A noteworthy observation concerning the force fields type used in both scoring functions is the usage of both physics and knowledge-based terms [27]. Since conformations produced by Rosetta only rely on short fragments, it has high flexibility in inferring new folds as clearly demonstrated by its state-of-the-art performance on FM targets in the latest CASP events [9,28-33].

Departing from Bowie and Eisenberg’s principles, but still considered as belonging to the fragment-assembly category, I-TASSER (Iterative Threading ASSEmbly Refinement) combines ab initio modelling and threading [7]. Since the length of the fragments chosen from threading has no upper limit (greater than or equal to 5), this method is suitable for both FM and template-based modelling (TBM) targets. As Rosetta, I-TASSER initially generates low resolution conformations, which are then refined. More specifically, structure prediction relies on three main stages [34]. First, sequence profile and predicted SS are used for threading through a representative set of the PDB. The highly-ranked template hits are selected for the next step. Second, structural assemblies are built using a coarse representation involving only C-alphas and centres of mass of the side chains. While fragments are extracted from the best aligned regions of the selected templates, pure ab initio modelling is used to create sections without templates. Fragment assemblies are performed by a modified version of the replica-exchange Monte Carlo simulation technique (REMC) [35] constrained by a knowledge-based force field including PDB-derived and threading constraints, and contact predictions. Generated conformations are then structurally clustered to produce a set of representatives, i.e. cluster centroids. Third, those structures are refined during another simulation stage to produce all-atom models. This mixed strategy has proved extremely successful since “Zhang-Server” [36], which is a combined pipeline of I-TASSER and QUARK (see next paragraph), has been ranked as the best server for protein structure prediction in the latest four CASP experiments (CASP7-10) [24,25], when all target categories are considered. However, when only FM targets associated with ab initio approaches are taken into account, Rosetta tends to provide more accurate models than I-TASSER [9,29,30,32].

Xu and Yang identified force fields and search strategies as the main limitations to accurate structure prediction [37]. They proposed a new approach, QUARK, which attempts to address them, while taking advantage of I-TASSER and Rosetta’s strengths. In addition to sequence profile and SS, QUARK also uses predicted solvent accessibility and torsion angles to select, like Rosetta and unlike I-TASSER, small fragments (size up to 20 residues) using a threading method for each sequence fragment. Then, using a semi-reduced model, i.e. the full backbone atoms and the side-chain centre of mass, and a variety of predicted structural features, an I-TASSER like pipeline is followed: assembly generation using REMC, conformation clustering and production of a few all-atom models. In this phase, not only does QUARK allow more conformational movements than I-TASSER, but also utilises a more advanced force field comprised of 11 terms including hydrogen bonding, SA and fragment-based distance profile, see [37] for details. When QUARK started contributing to CASP in its 9^th^ experiment, it was outperformed by Rosetta; however, positions were inverted in CASP10 [9,32].

All previously described fragment-based protein structure prediction methods are sequence-dependent since fragments are extracted from templates selected using sequence based information [16]. However, it has also been proposed to create databases of fragment models, which are chosen independently from their amino acid compositions to constitute conformation assemblies [38,39]. Fragments are only defined by their ‘shape’ and substituted in the query sequence at positions where amino acids can conform to those shapes. Although such techniques have not been competitive against sequence-dependent predictors, they have shown interesting results in modelling loops [38].

Although fragment assembly methods have been ranked as the most successful ones for free-modelling predictions, yet, many issues remain and need to be addressed [2]. First, successful attempts to produce accurate conformations have been mainly restricted to targets whose lengths are less than 100 residues [37] due to the enormous search space even though fragments are used instead of individual amino acids. Second, even for small proteins, processing time is prohibitive for the typical user; Rosetta, for instance, needs on average 150 CPU days per target [40]. Third, despite effective use of Monte Carlo simulations along with fragment replacements, a structure’s global minimum is likely to be missed. In addition, the design of the most appropriate force field is still a research question as current ones often fail to recognise native structure [8,37]. Finally, the large number of decoys produced by most of those methods constitutes an additional barrier to identification of native-like conformations since there is no straightforward correspondence between free energy values and similarity to a native structure. As a consequence, design of model quality assessment programs has become an active research area on its own [41,42].

As discussed, in twenty years, the field of fragment-based protein structure prediction has made very good progress, but there is still a lot of scope for improvement. A promising approach has been the integration within standard fragment-based systems of spatial constraints. So far, this has been performed using predicted contact maps [43,44]. Recently [45], integration of those constraints as a term into Rosetta’s energy function has led to significant improved model quality in terms of TM-score [46]. However, since accurate prediction of a contact map currently relies on the availability of a relatively large protein family (ideally more than 1000 homologous protein sequences) [47], their usage is not suitable for any protein target. Moreover, low quality contact maps lead invariably to poor models, since wrong constraints prevent exploration of the native structure conformation space. As a conclusion, there is a need for the design of alternative constraints to fragment-based protein structure prediction.

### Structural classification

Categorising protein structural classes was first introduced by Levitt and Chothia in 1976 [48] when proteins were found to belong to one of four classes: (1) all-alpha proteins; (2) all-beta proteins; (3) alpha + beta protein where beta strands tend to be segregated and likely to form antiparallel beta sheets; (4) alpha / beta proteins where alpha helices and beta strands are rather mixed and therefore polypeptide chains are expected to contain parallel beta sheets. Two decades later, Chothia et al. established a manually curated online database the Structural Classification Of Proteins (SCOP) [49]. The first level of its hierarchy was initially divided into five classes: the original four and a ‘multi-domain’ class. Later on two further classes were added, i.e. ‘Membrane and cell surface proteins and peptides’ and ‘Small proteins’ [50]. Despite this increase in class numbers, the original four classes still represent over 90% of all SCOP entries.

Two years after SCOP initial release, an alternative database, CATH – named after the first four levels of its hierarchy: Class, Architecture, Topology and Homology - was established [51]. Since they showed that there was no clear separation between alpha + beta and alpha/beta proteins [52,53], CATH has been based on only 4 classes: (1) mostly alpha; (2) mostly beta; (3) alpha beta and (4) Few secondary structures. Despite differences between SCOP and CATH, a comparative study [54] has shown the top level of both hierarchies, i.e. ‘Class’, is relatively consistent in comparison to the remaining levels since it is defined according to high level structural features.

**Figure 1:**
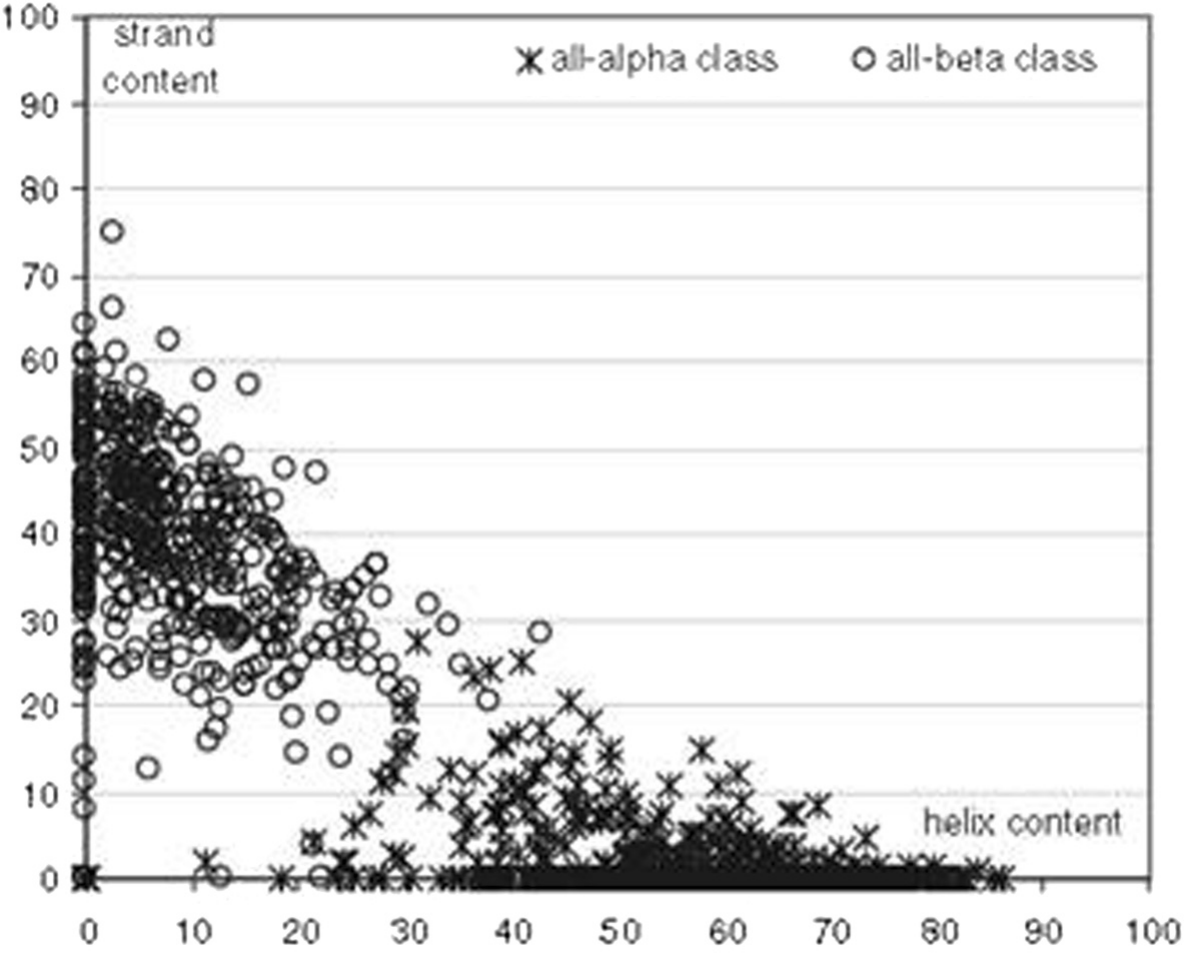
Scatter plot of helix and strand content (X-axis and Y-axis respectively) for a large set of proteins. Taken from: Kurgan LA, Zhang T, Zhang H, Shen S, Ruan J: Secondary structure-based assignment of the protein structural classes. *Amino Acids* 2008, 35:551–564. (With permission).

Assigning a protein structure to a specific class is not trivial. Whereas CATH uses an automated way [53], SCOP relies on manual inspection. Except for discrimination between ‘alpha/beta’ and ‘alpha + beta’, the critical criterion is the percentage of helix and strand contents. Many studies have been conducted to establish the best thresholds for classification, which led to a variety of values [55-62]. Eventually, a thorough comparative study, established that the 15% helix and 10% strand thresholds are optimal – those are used by CATH -, see Figure 1, even if overlapping regions exist between adjacent classes, especially ‘alpha/+beta’ and ‘mainly beta’ [55].

Since knowledge of a protein’s structural class from its sequence may reveal crucial information concerning folding types and functions [63,64] and can be considered as a first step towards solving structure prediction problem, sequence based class prediction has become an active research area [65]. Proposed approaches take advantage of either 1) machines learning techniques such as Support Vector Machines (SVM) [66-68], Artificial Neural Networks [69], rough sets [70], bagging [71], ensembles [72-75] and Meta-Classifiers [76,77] or 2) features that reveal class-related information like physiochemical-based information [73,78], pseudo amino acid composition [79,80], amino acid sequence reverse encoding [81,82], Position Specific Scoring Matrix (PPSM) profile [83] and structural based information including secondary structure prediction [55,84-86]. Detailed reviews can be found in [87,88]. Although state-of-the-art tools, including SCPRED [89], MODAS [81], RKS-PPSC [72], PSSS-PSSM [90], AADP-PSSM [91], SCEC [74], AATP [92], AAC-PSSM-AC [93] and PSSP-RFE [94] report overall accuracy that up to 90%, challenges remain in particular with proteins with low sequence similarity and discrimination between alpha/beta versus alpha + beta classes [90]. It is worth noting that most tools only deal with the four original SCOP classes which comprise around 90% of annotated domains [88].

### Overview

As highlighted in the review of fragment-based protein structure prediction approaches, their main limitation, as with all ab-initio methods, is their ability to sample efficiently the enormous protein configuration space which increases exponentially with protein sequence length. However, production of accurate predictions is eased if, for each given position, there is high proportion of fragments fitting closely the native one [95]: the higher the quality of the fragment libraries, the more focus the conformation search is on the sub-space containing the native structure. We propose to exploit this property by customising further fragment libraries according to the nature of the protein target. More specifically, we suggest tailoring the set of template proteins which are the source of those libraries so that their quality is increased. We formulate the hypothesis that protein structures that share structural information with a protein target are more likely to provide better fitting fragments than structurally unrelated proteins. Since sequence based structural class prediction has become relatively mature, we have decided to use such information to select the relevant template structures.

**Figure 2:**
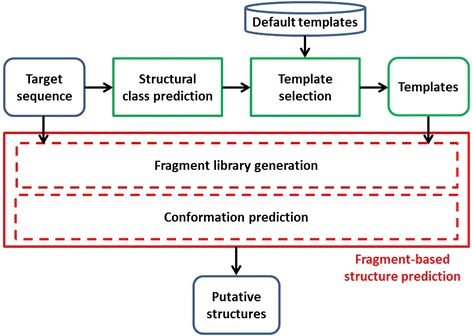
Proposed fragment-based protein structure prediction methodology.

From those principles, we have designed this new fragment-based protein structure prediction methodology, see Figure 2. First, structural class is predicted from the sequence of the protein target. Second, a target specific list of template structures is generated by extracting high resolution templates sharing the same structural class from the default template protein set (a PDB subset) associated to the fragment-based method. Finally, the target sequence and its associated template list are submitted to a fragment-based protein structure prediction, which produces customised fragment libraries and generates a set of putative structures of the protein target.

In this paper, we conduct an exhaustive evaluation of our methodology on a set of recent CASP targets. First, we compare the quality of models with and without class annotations, including the case when structural classes are predicted from sequence. Second, we analyse the influence of the class type on structure prediction performance. Third, we study the impact of class annotations in terms of convergence towards the best conformation. Fourth, we discuss the validity of the proposed methodology and its potential application. Finally, we provide a detailed presentation of the proposed fragment-based protein structure prediction methodology.

## Results

### Dataset, databases and software tools

The target dataset comprises 70 proteins selected from the latest CASP contests. First, only proteins containing fewer than 150 amino acids were considered since larger targets would show a complexity which is generally believed to be beyond the capabilities of state-of-the-art ab initio methods [7]. Second, the selection process aimed at producing a set of FM targets showing diversity in terms of structural class. However, in order to be able to produce statistically significant results, the initial set was extended using TBM targets. In any case, the experimental protocol was designed so that predictions would be made independently of the presence of homologous structures in the template set.

In terms of structural class prediction, the two main classifications, i.e. CATH [96] and SCOP [97], were considered. Class annotations used in experiments were collected from two sources: annotations based on actual protein structures – which are treated as the gold standard - and sequence based predictions performed by MODAS [79]. Finally, structure prediction was performed using the fragment based de novo protein structure prediction software offered by the Rosetta suite [98], where the number of selected fragments for each position was left to its default value, i.e. 200. In order to cover a reasonably high number of permutations amongst the total number of fragments, Rosetta’s team recommends generating between 20,000 and 30,000 models [12]. Therefore, we decided to generate 20,000 conformations for each experiment to conduct a thorough study. Their evaluation was performed using both the GDT_TS (GDT in the text) and RMSD metrics of the 10 highest and lowest models respectively.

### General performance

First, quality of the models generated by the standard Rosetta framework, i.e. without using any structural class annotation, is compared to those produced using the gold standard, i.e. structure based, class annotations. As Table 1 shows, average performance for the 70 targets (target specific results are shown in Additional file 1: Table S1) in terms of both RMSD and GDT demonstrates that class annotation allows better structure prediction (~6% improvement). Those differences are statistically highly significant since p-values < 0.0005 and < 0.005, respectively. On the other hand, there is no significant difference between the SCOP and CATH based approaches in terms of both GDT and RMSD (p-values > 0.05).

**Table 1:** **Average performance (and standard deviation) in terms of GDT and RMSD, and associated p-values**

	**No class annotation**	**CATH class annotation**		**SCOP class annotation**	
		**Structure based**	**Sequence based (MODAS predictions)**	**Structure based**	**Sequence based (MODAS predictions)**
GDT	46.04 (13.89)	48.62 (14.22) p = 0.00007	47.64 (14.10)	48.92 (14.97) p = 0.0002	48.31 (15.14)
RMSD	6.4 (2.3)	6.0 (2.2) p = 0.0005	6.1 (2.2)	6.0 (2.3) p = 0.004	6.1 (2.3)

Sequence based annotations are the one taken from MODAS predictions. GDT and RMSD are the average of the GDT_TS and RMSD of the 70 targets, which in turn, are the average of the highest and lowest 10 scores respectively.

In addition, Table 1 reveals that predictions based on MODAS automatic annotations are only marginally worse than those based on structure based class annotations especially for SCOP. This can be explained, first, by the very good accuracy of MODAS predictions and, second, by the fact that misclassifications only appear between classes with blurred borders [53]. Comparison between structure and sequence-based annotations shows that 78.5% and 81.4% of classes have been correctly predicted by MODAS for SCOP and CATH respectively. As expected, there is higher accuracy for CATH since there is no differentiation between alpha/beta and alpha + beta classes. Indeed, the confusion matrix shown in Table 2 highlights that confusion only occurs between alpha and alpha_beta, or beta and alpha_beta, or FSS and alpha_beta classes (differences in the latter case happen since targets lie on the border between those classes, see Additional file 1: Table S1), but never between alpha and beta classes. Those results demonstrate that usage of a structural class predictor makes our pipeline practical and allows the generation of better models than those produced by the standard Rosetta framework. Since structural class prediction is an active research area, there is no doubt that performance obtained with predicted classes will get even closer to those attained with actual classes in the near future. Given that the aim of this paper is to demonstrate and analyse the value of fragment libraries generated from class specific templates, the remaining analysis concentrates on results generated from structure-based class annotations.

**Table 2:** **Confusion matrix showing CATH classes versus MODAS predicted ones**

**Predicted gold standard**	**A**	**A_B**	**B**	**FSS**
A	15	1	0	0
A_B	2	25	3	3
B	0	4	14	0
FSS	0	0	0	3

**Figure 3:**
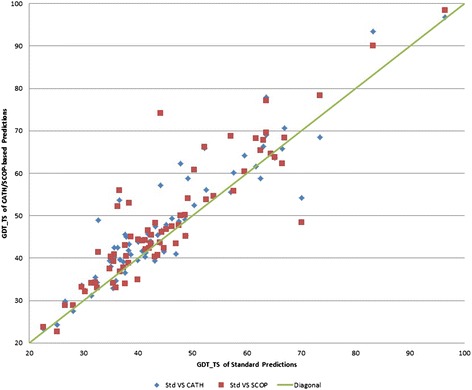
GDT of standard predictions versus CATH and SCOP-based predictions for the 70 targets.

**Figure 4:**
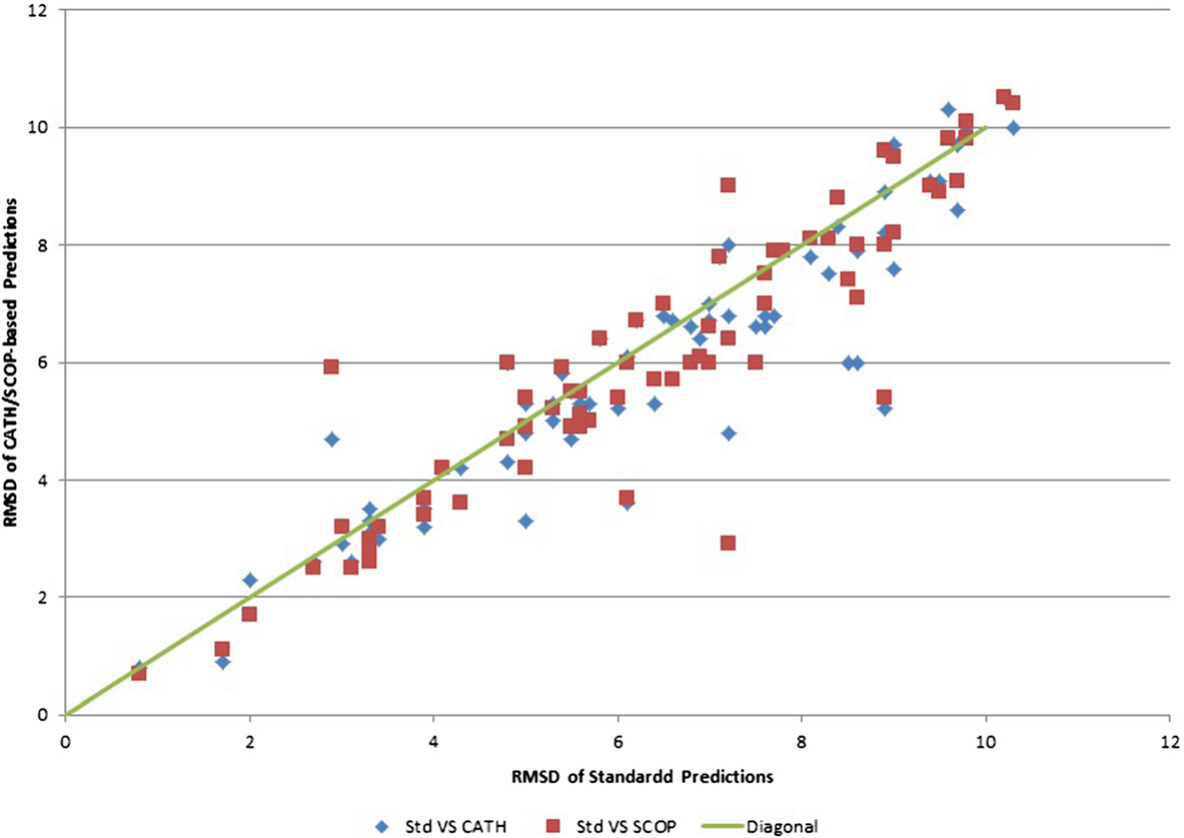
RMSD of standard predictions versus CATH and SCOP-based predictions for the 70 targets.

As Figures 2 and 3 show, predictions based on structural class annotations outperform standard ones for a majority of targets. Actually, higher GDT is obtained for 70.0% and 78.6% of the targets using CATH and SCOP respectively (Figure 3), whereas better RMSD is shown for 61.4% and 67.1% of the targets (Figure 4). More detailed information is shown in Table 3, whereas target specific data are provided in Additional file 1: Table S1.

**Table 3:** **Performance comparison for the 70 targets**

	**Metric**	**Percentage of improved targets (average change)**	**Percentage of unaffected targets**	**Percentage of worsened targets (average change)**
CATH	GDT	70.00% (+4.77, i.e. +11.19%)	0.00%	30.00% (−2.53, i.e. -4.83%)
	RMSD	61.43% (+0.81, i.e. +12.86%)	11.42%	27.15% (−0.49, i.e. -10.09%)
SCOP	GDT	78.57% (+4.77, i.e. +10.98%)	0.00%	21.43% (−4.01, i.e. -8.07%)
	RMSD	67.15% (+0.73, i.e. +12.45%)	4.28%	28.57% (−0.61, i.e. -12.34%)

Numbers are extracted and analysed from the Additional file 1: Table S1 for the whole dataset.

### Performance according to structural class

Since SCOP and CATH-based produces similar results, we can conclude that those classifications are equally informative in terms of protein template selection; however, that may not be case for all classes. Hence, we have conducted a more in depth analysis by focusing on performance enhancement according to the structural class of the target (see Table 4). First, whatever the classification, targets from all main classes benefit significantly from template selection: the number of targets with models displaying a better GDT is between 61.1% and 100.0%. Interestingly, targets combining Alpha and Beta structures seem to gain more from the proposed methodology. One may suggest that, since structural discontinuities between secondary structure elements are key to a protein conformation, using libraries with a higher content of alpha to/from beta transition fragments leads to better conformation predictions.

**Table 4:** **Performance comparison according to structural class**

	**CATH-based predictions**			**SCOP-based predictions**		
**Targets**	**Class (Total # of templates)**	**Targets with better GDT**	**Targets with both better GDT & RMSD**	**Class (Total # of templates)**	**Targets with better GDT**	**Targets with both better GDT & RMSD**
16	Mainly Alpha (10194)	75.0%	62.5%	All Alpha (4807)	75.0%	56.3%
18	Mainly Beta (10532)	61.1%	38.9%	All Beta (7534)	77.8%	55.6%
33 (29+ 4)	Alpha Beta (22685)	75.8%	63.6%	Alpha + Beta (7824)	86.2%	65.6%
				Alpha / Beta (9186)	100.0%	100.0%
3	Few Secondary Structures (531)	33.3%	0.0%	Small Proteins (853)	66.6%	66.6%
70	All	68.6%	54.3%	All	81.4%	62.9%

Second, as expected, association to less common classes that are not specific in terms of structural content, i.e. Few Secondary Structures (FSS) and Small Proteins (SP), seem to be less beneficial with (SP) or even detrimental (FSS) to structure prediction. Although one should be cautious when discussing results for such a small number of targets, the fact that the number of templates associated with those classes is a degree of magnitude lower than the main classes’ may also lead to the generation of fragment libraries which do not cover sufficiently the conformation space. Third, except for the ‘Alpha’ class, where CATH class annotations contribute to slightly better results, SCOP’s lead to a marginally higher number of targets with improved models (see Table 3 for details). One can also note that, except in the case of SP and FSS classes where it is very low, the number of templates does not seem to impact on structure prediction.

### Convergence towards native-like conformations

**Figure 5:**
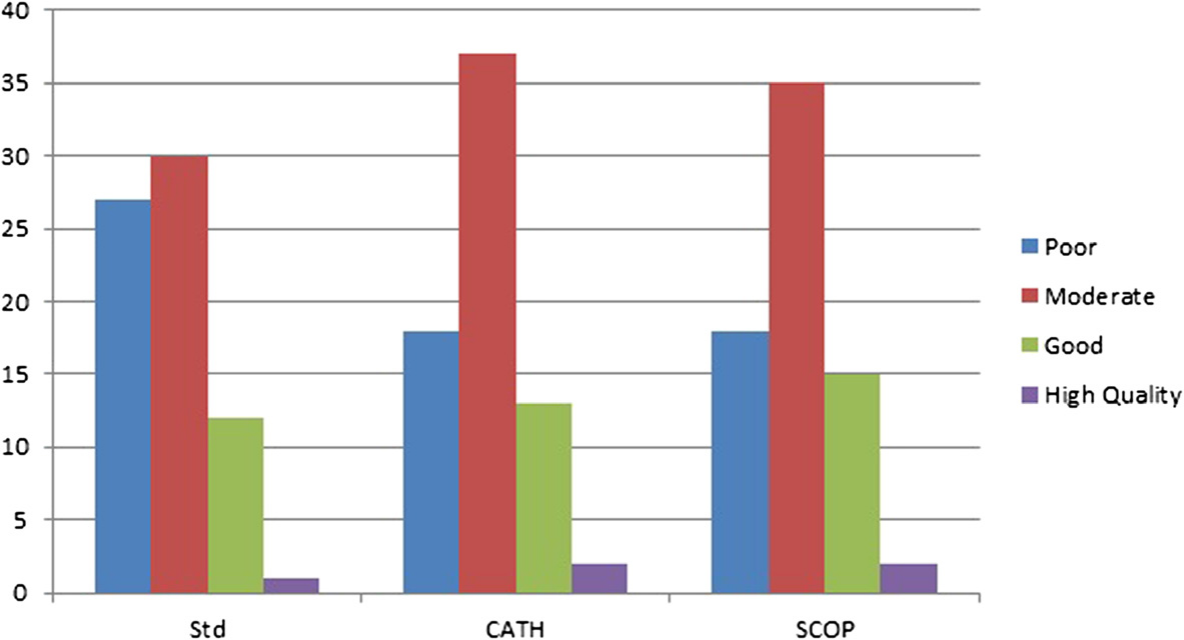
Qualitative distribution of the average GDT of the best 10 models.

Although we have shown that methods relying on structural class-based libraries generally generate better conformations than the standard Rosetta framework, it is important to know if this leads to a notable change in terms of model significance. To address this question, we performed classification of the average of the best 10 model for each target according to thresholds adopted in the literature. Production of models the GDT of which are above 40 is particularly important since their conformation is believed to have the same ‘shape’ as the target, which may reveal crucial information about potential proteins’ functions [99,100]. Models whose GDT value is greater than or equal to 85 are judged convenient to solve the phase problem in crystallography [101]. Conformations with GDT higher than 59 are believed to be’good‘enough [102], whilst structures with GDT lower than 40 are considered of poor quality or even random [103,104]. Consequently, we will adopt the following thresholds and associated classes: “Poor” for GDT < 40, “Moderate” for GDT between 40 and 59, “Good” between 60 and 84, and “High Quality” for GDT > 84. As Figure 5 shows, whereas the standard Rosetta framework is able to produce informative models for 61.4% of the targets, both SCOP and CATH-based schemes deliver a much larger proportion of them, 74.8% for both.

Since part of the rational of the proposed methodology is a reduction of the size of the conformation space, we calculated for each target the number of conformations which were generated in order to produce the structure with highest GDT or/and lowest RMSD out of the 20,000. SCOP and CATH-based experiments produce both their best GDT and RMSD structures after generating a smaller number of conformations than the standard Rosetta framework, converging towards those conformations, respectively, 2.8% and 6.9% faster (see Table 5). In addition, since correlation between GDT and RMSD increases when conformations are getting closer to the native one, the generation of models which display both the Highest GDT and the Lowest RMSD indicate that a predictor tends to produce more native-like conformations. Out of the 70 targets, 9, 10 and 16 protein conformations share best GDT and RMSD in experiments conducted using the standard Rosetta framework, SCOP and CATH classes, respectively. Although both SCOP and CATH classes allow generation of more of those models, this is particularly significant for CATH outputs since there is an increase of 78% compared to the standard Rosetta framework.

**Table 5:** **Average number of conformations for convergence towards the structure with highest GDT or/and lowest RMSD (and associated standard deviations)**

	**Standard predictions**	**SCOP-based predictions**	**CATH-based predictions**
GDT	10848 (5469)	9743 (5753)	9452 (5968)
RMSD	9836 (5536)	10166 (5770)	10491 (5639)
GDT & RMSD	13560 (4707)	13175 (4583)	12625 (5125)

## Discussion

Following an exhaustive evaluation of our methodology, we have demonstrated that usage of class annotations leads to highly significant enhanced structure prediction performance (p-values < 0.005), even if they have been predicted from sequence alone. Although experiments were conducted using two different types of structural classifications, i.e. CATH and SCOP, there is no convincing evidence suggesting that one is more appropriate than the other. Performance analysis according to structural type class shows that targets from all main and well defined classes benefit from the proposed methodology. Moreover, quality of structure prediction does not appear to be influenced by the number of selected template, if it is above a few 1000s. All these results support our hypothesis that template quality in terms of structural relevance is more important than quantity and diversity. In addition, experiments conducted using structural class prediction demonstrates the proposed methodology is practical.

Further results analysis also shows that methods relying on class-based libraries produce conformations which are more relevant to user, i.e. more ‘good’ and ‘accurate’ models. In addition, since structure predictors converge quicker towards the best model, this substantiates our claim that usage of structurally relevant templates conduct to reducing the size of the conformation space to be explored.

## Conclusions

In this paper, we have proposed usage of structural class constraints for ab initio fragment-based protein structure prediction to decrease the size of the conformation search space. Then, using Rosetta, a comprehensive evaluation of our methodology has been conducted on a set of recent CASP targets. We have demonstrated that exploitation of class annotations leads to enhanced structure prediction performance; even if they are predicted since current sequence based predictions are sufficiently accurate. Results also support our hypothesis that reduction towards a better focused structure space conducts to quicker identification of better models.

Since our methodology produces models the quality of which is up to 7% higher in average than those generated by a standard fragment-based predictor, we believe it should be considered before conducting any fragment-based protein structure prediction. Despite such progress, ab initio prediction remains a challenging task, especially for proteins of average and large sizes. Apart from improving search strategies and energy functions, integration of additional constraints seems a promising route, especially if they can be accurately predicted from sequence alone.

## Methods

### Fragment-based protein structure prediction software

Since we propose to enhance performance of fragment-based protein structure predictors by customising their fragment libraries, validation relies on using an existing predictor which can be tailored to suit our methodology. Among state-of-the-art methods, QUARK does not provide user control of protein template selection and it has only been available very recently for I-TASSER (V4.1 released in August 2014). As a consequence, Rosetta was selected, since, in addition to offer state-of-the-art ab initio protein structure predictions [9], it is open-source, providing full control of the template proteins used for fragment extraction [98].

In Rosetta, fragment-based protein structure prediction relies on high resolution template proteins to excise fragments from. When using the standard Rosetta framework, the database of template proteins of Rosetta’s web server is used [105]. Indeed, Rosetta’s developers strongly recommend using it since it is supposed to contain idealised and diverse collections of structures that are believed to allow the construction of any possible conformation. However, the Rosetta package also offers the facility – a local fragment builder called ‘Fragment_Picker’ [106] and a local copy of the database of template proteins called “vall” - to build user-specific fragment libraries by using a user-defined set of templates.

Here, our approach takes advantage of that capacity under the ‘Quota’ protocol, which is specifically designed for ab initio predictions, so that the high resolution template proteins selected by structural class annotation of the target become the source of the fragment libraries. We have used the latest version of the “vall” supported by Rosetta3, which comprises high resolved proteins of different classes and folds. A list of a class’s PDB code is provided to “Fragment_Picker”, so that the intersection of that set and “vall” is used as fragment libraries’ source.

### Structural class annotations

Our novel approach relies on structural class annotations of target sequences. Both SCOP and CATH are widely used databases, attracting diverse publics according to appreciation of their different degrees of automation. Since SCOP-based annotations rely largely on a manual process, they are preferred by many biologists as it is seen to be “more natural” [55]. On the other hand, CATH’s higher degree of automation makes annotations more systematic and allows processing a larger share of the PDB. Here both classification schemes are considered in our evaluation. Since we wish to both validate the concept of using class-specific fragment libraries for protein structure predictions and demonstrate its practicality, all protein targets were annotated twice based on either their known structure – classifications seen as the gold standard - or their sequence.

First, structural class annotations, according to both SCOP and CATH classifications, were conducted on all protein targets using their structure. Note that all selected targets only contained a single domain. Initially, when available, annotations were extracted from SCOP and CATH databases. If a target was present only in one of the two, the second annotation could generally be deduced directly. However, in the case of a protein belonging to CATH’s class ‘alpha beta’, manual inspection was used to allocate it to either the alpha/beta or alpha + beta class in the SCOP classification. Alternatively, when targets did not have any annotation in neither databases, we classified them manually based on the secondary structure contents of their PDB entry as provided by the Dictionary of Protein Secondary Structure (DSSP) [107] and the thresholds adopted by CATH [53].

Second, class annotations were predicted from sequence alone. As seen in the ‘Background’ section, structural class prediction is a very mature field where accuracy reaches up to 90%. Among the most competitive methods, MODAS [79] - MODular Approach to Structural class prediction – is particularly suitable for our application since it is freely available online and it provides predictions for the main seven classes of SCOP, from which CATH-like annotations can automatically inferred. MODAS classifiers are based on a SVM which operates on combined features from both predicted secondary structure and multiple sequence alignment profiles.

### Evaluation framework

In order to evaluate the proposed framework, predictions have to be performed using protein sequences the structure of which is known. Since we intend to simulate ab initio protein structure prediction, it is important to make sure that information about the actual native and potential homologous structures is not exploited. As a consequence, when the standard Rosetta framework is used the ‘exclude homologues’ flag is set, whereas the pipeline presented in Figure 2 was slightly modified.

First, structural class annotation is conducted according to the experiment aim, i.e. concept validation or practicality demonstration using either CATH or SCOP. Second, all high quality structures of the PDB belonging to same structural class are extracted. A 2.5 Angstrom resolution cut-off is used to produce high quality fragments. Third, the target and all its homologues (based on PSI-BLAST with an E-value < 0.05) were removed from the set of collected structures. Fourth, the fragment libraries were constructed by providing Rosetta’s fragment-picker with this set of protein templates. Apart from setting the ‘exclude homologues’ flag, all the default options were kept including parameter weights and the number of fragments at each position, i.e. 200. Finally, since picking and assembling fragments to construct a whole conformation is a stochastic process that relies on Monte Carlo simulation, it needs to be performed a large number of times. As it is suitable to produce as many as possible structures for each target as an attempt to cover the highest number of permutations amongst the total number of fragments, the recommended value of 20,000 models was chosen for all experiments [12].

### Evaluation metrics

The main metric used to assess our structure prediction pipeline is the global distance test-total score (GDT_TS). It was introduced as a part of the LGA (Local Global Alignment) method and since then it has been widely accepted in the community mainly due the fact it is less sensitive to outliers than the popular root mean square deviation (RMSD) [108]. GDT_TS is the formal criterion CASP uses in order to qualify and assess Tertiary Structure (TS) prediction and it is defined as the average of the percentage of residues that are less than 1, 2, 4, and 8 angstroms. For the sake of completeness, we have also included the RMSD in our analysis. Metrics were generated using MaxCluster, a tool for protein structure comparison and clustering [109]. Since our study mainly aims at improving the quality of the generated conformations, structure results are evaluated using the average of the best 10 scores for each metric, although results for the best score of each metric are provided as well in the Additional file 1: Table S1. Therefore, whenever GDT and RMSD are mentioned in this paper, unless otherwise stated, they refer to the average of the highest 10 GDT_TS and lowest 10 RMSD respectively. Besides, GDT_TS and RMSD, GDT-HA (High Accuracy) is also shown in the detailed results presented in the Additional file 1: Table S1 since it proves useful especially for high accuracy predictions. It is defined as the average of the percentage of residues that superimpose within 0.5, 1, 2, and 4 angstroms.

## Additional files

Additional file 1: Table S1. It includes the detailed results for the 70 targets using three metrics: GDT_TS, GDT_HA and RMSD for the three experiments (Standard, CATH-based and SCOP-based). For each experiment two sets of data are provided; the best and the average of the best 10 scores of each metric.

## Abbreviations

SCOP: Structural classification of proteins
CATH: Class, architecture, topology, and homologous superfamily
FSS: Few secondary structures
SP: Small proteins
MODAS: MODular approach to structural class prediction
SVM: Support vector machine
DSSP: Dictionary of secondary structure of proteins
PDB: Protein data bank

## References

[1] Kendrew JC, Bodo G, Dintzis HM, Parrish RG, Wyckoff H, Philips DC (1958). A three-dimensional model of the myoglobin molecule obtained by x-ray analysis. Nature.

[2] Dill KA, MacCallum JL (2012). The protein-folding problem, 50 years on. Science.

[3] Anfinsen CB, Haber E, Sela M, White FH (1961). The kinetics of formation of native ribonuclease during oxidation of the reduced polypeptide chain. Proc Natl Acad Sci U S A.

[4] Lee J, Liwo A, Ripoll DR, Pillardy J, Saunders JA, Gibson KD (2000). Hierarchical energy-based approach to protein-structure prediction: Blind-test evaluation with CASP3 targets. Int J Quantum Chem.

[5] Shaw DE, Maragakis P, Lindorff-Larsen K, Piana S, Dror RO, Eastwood MP (2010). Atomic-level characterization of the structural dynamics of proteins. Science.

[6] Lindorff-Larsen K, Piana S, Dror RO, Shaw DE (2011). How fast-folding proteins fold. Science.

[7] Abbass J, Nebel J-C, Mansour N, Elloumi M, Zomaya AY (2013). Ab Initio Protein Structure Prediction: Methods and challenges. Biol Knowl Discov Handb.

[8] Lee J, Wu S, Zhang Y (2009). Ab initio protein structure prediction. From Protein Structure to Function with Bioinformatics.

[9] Tai CH, Bai H, Taylor TJ, Lee B (2014). Assessment of template-free modeling in CASP10 and ROLL. Proteins.

[10] Lu W, Liu H (2007). Correlations Between Amino Acids at Different Sites in Local Sequences of Protein Fragments with Given Structural Patterns. Chin J Chem Phys.

[11] Bowie JU, Eisenberg D (1994). An evolutionary approach to folding small alpha-helical proteins that uses sequence information and an empirical guiding fitness function. Proc Natl Acad Sci U S A.

[12] Bradley P, Misura KMS, Baker D (2005). Toward high-resolution de novo structure prediction for small proteins. Science.

[13] Hockenmaier J, Joshi AK, Dill KA (2007). Routes are trees: the parsing perspective on protein folding. Proteins.

[14] Voelz VA, Dill KA (2007). Exploring zipping and assembly as a protein folding principle. Proteins.

[15] Bystroff C, Simons KT, Han KF, Baker D (1996). Local sequence-structure correlations in proteins. Curr Opin Biotech.

[16] Xu D, Zhang Y (2013). Toward optimal fragment generations for ab initio protein structure assembly. Proteins.

[17] Jones DT (1997). Successful ab initio prediction of the tertiary structure of NK-lysin using multiple sequences and recognized supersecondary structural motifs. Proteins.

[18] Moult J, Pedersen JT, Judson R, Fidelis K (1995). A large-scale experiment to assess protein structure prediction methods. Proteins.

[19] Jones DT, Bryson K, Coleman A, McGuffin LJ, Sadowski MI, Sodhi JS (2005). Prediction of novel and analogous folds using fragment assembly and fold recognition. Proteins.

[20] Wright PE, Dyson HJ, Lerner RA (1988). Conformation of peptide fragments of proteins in aqueous solution: implications for initiation of protein folding. Biochemistry.

[21] Dyson HJ, Sayre JR, Merutka G, Shin HC, Lerner RA, Wright PE (1992). Folding of peptide fragments comprising the complete sequence of proteins. Models for initiation of protein folding. II. Plastocyanin. J Mol Biol.

[22] Jones DT (2001). Predicting novel protein folds by using FRAGFOLD. Proteins.

[23] Jones DT, McGuffin LJ (2003). Assembling novel protein folds from super-secondary structural fragments. Proteins.

[24] Schonbrun J, Wedemeyer WJ, Baker D (2002). Protein structure prediction in 2002. Curr Opin Struct Biol..

[25] Han KF, Baker D (1996). Global properties of the mapping between local amino acid sequence and local structure in proteins. Proc Natl Acad Sci U S A.

[26] Simons KT, Kooperberg C, Huang E, Baker D (1997). Assembly of protein tertiary structures from fragments with similar local sequences using simulated annealing and Bayesian scoring functions. J Mol Biol.

[27] Rohl CA, Strauss CEM, Misura KMS, Baker D (2004). Protein structure prediction using Rosetta. Methods Enzymol.

[28] Vincent JJ, Tai C-H, Sathyanarayana BK, Lee B (2005). Assessment of CASP6 predictions for new and nearly new fold targets. Proteins.

[29] Jauch R, Yeo HC, Kolatkar PR, Clarke ND (2007). Assessment of CASP7 structure predictions for template free targets. Proteins.

[30] Ben-David M, Noivirt-Brik O, Paz A, Prilusky J, Sussman JL, Levy Y (2009). Assessment of CASP8 structure predictions for template free targets. Proteins.

[31] Bradley P, Malmstrom L, Qian B, Schonbrun J, Chivian D, Kim DE, Meiler J, Misura KM, Baker D (2005). Free modeling with Rosetta in CASP6. Proteins..

[32] Kinch L, Yong Shi S, Cong Q, Cheng H, Liao Y, Grishin NV (2011). CASP9 assessment of free modeling target predictions. Proteins.

[33] Raman S, Vernon R, Thompson J, Tyka M, Sadreyev R, Pei J (2009). Structure prediction for CASP8 with all-atom refinement using Rosetta. Proteins.

[34] Roy A, Kucukural A, Zhang Y (2010). I-TASSER: a unified platform for automated protein structure and function prediction. Nat Protoc.

[35] Zhang Y, Kihara D, Skolnick J (2002). Local energy landscape flattening: Parallel hyperbolic Monte Carlo sampling of protein folding. Proteins.

[36] Zhang Y (2014). Interplay of I-TASSER and QUARK for template-based and ab initio protein structure prediction in CASP10. Proteins.

[37] Xu D, Zhang Y (2012). Ab initio protein structure assembly using continuous structure fragments and optimized knowledge-based force field. Proteins.

[38] Kolodny R, Koehl P, Guibas L, Levitt M (2002). Small libraries of protein fragments model native protein structures accurately. J Mol Biol.

[39] Baeten L, Reumers J, Tur V, Stricher F, Lenaerts T, Serrano L (2008). Reconstruction of protein backbones from the BriX collection of canonical protein fragments. PLoS Comput Biol.

[40] Wu S, Skolnick J, Zhang Y (2007). Ab initio modeling of small proteins by iterative TASSER simulations. BMC Biol.

[41] Konopka BM, Nebel J-C, Kotulska M (2012). Quality assessment of protein model-structures based on structural and functional similarities. BMC Bioinformatics.

[42] Cao R, Wang Z, Wang Y, Cheng J (2014). SMOQ: a tool for predicting the absolute residue-specific quality of a single protein model with support vector machines. BMC Bioinformatics.

[43] Wu S, Szilagyi A, Zhang Y (2011). Improving protein structure prediction using multiple sequence-based contact predictions. Structure.

[44] Kosciolek T, Jones DT (2014). De novo structure prediction of globular proteins aided by sequence variation-derived contacts. PLoS One.

[45] Michel M, Hayat S, Skwark MJ, Sander C, Marks DS, Elofsson A (2014). PconsFold: improved contact predictions improve protein models. Bioinformatics.

[46] Zhang Y, Skolnick J (2005). TM-align: a protein structure alignment algorithm based on the TM-score. Nucleic Acids Res.

[47] Skwark MJ, Raimondi D, Michel M, Elofsson A (2014). Improved Contact Predictions Using the Recognition of Protein Like Contact Patterns. PLoS Comput Biol.

[48] Levitt M, Chothia C (1976). Structural patterns in globular proteins. Nature.

[49] Murzin AG, Brenner SE, Hubbard T, Chothia C (1995). SCOP: a structural classification of proteins database for the investigation of sequences and structures. J Mol Biol.

[50] Lo Conte L, Brenner SE, Hubbard TJP, Chothia C, Murzin AG (2002). SCOP database in 2002: refinements accommodate structural genomics. Nucleic Acids Res.

[51] Orengo CA, Michie AD, Jones S, Jones DT, Swindells MB, Thornton JM (1997). CATH–a hierarchic classification of protein domain structures. Structure.

[52] Berman HM, Westbrook J, Feng Z, Gilliland G, Bhat TN, Weissig H (2000). The Protein Data Bank. Nucleic Acids Res.

[53] Michie AD, Orengo CA, Thornton JM (1996). Analysis of domain structural class using an automated class assignment protocol. J Mol Biol.

[54] Csaba G, Birzele F, Zimmer R (2009). Systematic comparison of SCOP and CATH: a new gold standard for protein structure analysis. BMC Struct Biol.

[55] Kurgan LA, Zhang T, Zhang H, Shen S, Ruan J (2008). Secondary structure-based assignment of the protein structural classes. Amino Acids.

[56] Nakashima H, Nishikawa K, Ooi T (1986). The folding type of a protein is relevant to the amino acid composition. J Biochem.

[57] Klein P, Delisi C (1986). Prediction of protein structural class from the amino acid sequence. Biopolymers.

[58] Chou P, Fasman G (1989). Prediction of Protein Structural Classes from Amino Acid Compositions. Prediction of Protein Structural Classes from Amino Acid Compositions - 12.

[59] Kneller DG, Cohen FE, Langridge R (1990). Improvements in protein secondary structure prediction by an enhanced neural network. J Mol Biol.

[60] Chou KC (1995). A novel approach to predicting protein structural classes in a (20–1)-D amino acid composition space. Proteins.

[61] Eisenhaber F, Frömmel C, Argos P (1996). Prediction of secondary structural content of proteins from their amino acid composition alone. II The paradox with secondary structural class. Proteins.

[62] Chou KC, Liu WM, Maggiora GM, Zhang CT (1998). Prediction and classification of domain structural classes. Proteins.

[63] Chou KC (2005). Using amphiphilic pseudo amino acid composition to predict enzyme subfamily classes. Bioinformatics.

[64] Chou KC, Zhang CT (1995). Prediction of protein structural classes. Crit Rev Biochem Mol Biol.

[65] Chou KC (2011). Some remarks on protein attribute prediction and pseudo amino acid composition. J Theor Biol..

[66] Dehzangi A, Paliwal K, Lyons J, Sharma A, Sattar A (2014). Proposing a highly accurate protein structural class predictor using segmentation-based features. BMC Genomics.

[67] Anand A, Pugalenthi G, Suganthan PN (2008). Predicting protein structural class by SVM with class-wise optimized features and decision probabilities. J Theor Biol.

[68] Hayat M, Khan A (2012). Mem-PHybrid: Hybrid features-based prediction system for classifying membrane protein types. Anal Biochem.

[69] Jahandideh S, Abdolmaleki P, Jahandideh M, Asadabadi EB (2007). Novel two-stage hybrid neural discriminant model for predicting proteins structural classes. Biophys Chem.

[70] Cao Y, Liu S, Zhang L, Qin J, Wang J, Tang K (2006). Prediction of protein structural class with Rough Sets. BMC Bioinformatics.

[71] Dong L, Yuan Y, Cai Y (2006). Using Bagging classifier to predict protein domain structural class. J Biomol Struct Dyn.

[72] Yang J-Y, Peng Z-L, Chen X (2010). Prediction of protein structural classes for low-homology sequences based on predicted secondary structure. BMC Bioinformatics.

[73] Dehzangi A, Paliwal K, Sharma A, Dehzangi O, Sattar A (2013). A combination of feature extraction methods with an ensemble of different classifiers for protein structural class prediction problem. IEEE/ACM Trans Comput Biol Bioinform.

[74] Chen KE, Kurgan LA, Ruan J (2008). Prediction of protein structural class using novel evolutionary collocation-based sequence representation. J Comput Chem.

[75] Hayat M, Khan A, Yeasin M (2012). Prediction of membrane proteins using split amino acid and ensemble classification. Amino Acids.

[76] Cai YD, Feng KY, Lu WC, Chou KC (2006). Using LogitBoost classifier to predict protein structural classes. J Theor Biol.

[77] Feng KY, Cai YD, Chou KC (2005). Boosting classifier for predicting protein domain structural class. Biochem Biophys Res Commun.

[78] Li Z-C, Zhou X-B, Lin Y-R, Zou X-Y (2008). Prediction of protein structure class by coupling improved genetic algorithm and support vector machine. Amino Acids.

[79] Chou KC (2000). Prediction of protein structural classes and subcellular locations. Curr Protein Pept Sci.

[80] Ding Y-S, Zhang T-L, Chou K-C (2007). Prediction of protein structure classes with pseudo amino acid composition and fuzzy support vector machine network. Protein Pept Lett.

[81] Mizianty MJ, Kurgan L (2009). Modular prediction of protein structural classes from sequences of twilight-zone identity with predicting sequences. BMC Bioinformatics.

[82] Deschavanne P, Tufféry P (2008). Exploring an alignment free approach for protein classification and structural class prediction. Biochimie.

[83] Hayat M, Khan A (2012). MemHyb: Predicting membrane protein types by hybridizing SAAC and PSSM. J Theor Biol.

[84] Liu T, Jia C (2010). A high-accuracy protein structural class prediction algorithm using predicted secondary structural information. J Theor Biol.

[85] Kurgan L, Chen K (2007). Prediction of protein structural class for the twilight zone sequences. Biochem Biophys Res Commun.

[86] Jones DT (1999). Protein secondary structure prediction based on position-specific scoring matrices. J Mol Biol.

[87] Kurgan LA, Homaeian L (2006). Prediction of structural classes for protein sequences and domains-Impact of prediction algorithms, sequence representation and homology, and test procedures on accuracy. Pattern Recogn.

[88] Chou K-C (2005). Progress in protein structural class prediction and its impact to bioinformatics and proteomics. Curr Protein Pept Sci.

[89] Kurgan L, Cios K, Chen K (2008). SCPRED: accurate prediction of protein structural class for sequences of twilight-zone similarity with predicting sequences. BMC Bioinformatics.

[90] Ding S, Li Y, Shi Z, Yan S (2014). A protein structural classes prediction method based on predicted secondary structure and PSI-BLAST profile. Biochimie.

[91] Liu T, Zheng X, Wang J (2010). Prediction of protein structural class for low-similarity sequences using support vector machine and PSI-BLAST profile. Biochimie.

[92] Zhang S, Ye F, Yuan X (2012). Using principal component analysis and support vector machine to predict protein structural class for low-similarity sequences via PSSM. J Biomol Struct Dyn.

[93] Liu T, Geng X, Zheng X, Li R, Wang J (2012). Accurate prediction of protein structural class using auto covariance transformation of PSI-BLAST profiles. Amino Acids.

[94] Li L, Cui X, Yu S, Zhang Y, Luo Z, Yang H (2014). PSSP-RFE: Accurate prediction of protein structural class by recursive feature extraction from PSI-BLAST profile, physical-chemical property and functional annotations. PLoS One..

[95] Handl J, Knowles J, Vernon R, Baker D, Lovell SC (2012). The dual role of fragments in fragment-assembly methods for de novo protein structure prediction. Proteins.

[96] Sillitoe I, Cuff AL, Dessailly BH, Dawson NL, Furnham N, Lee D (2013). New functional families (FunFams) in CATH to improve the mapping of conserved functional sites to 3D structures. Nucleic Acids Res.

[97] Andreeva A, Howorth D, Chothia C, Kulesha E, Murzin AG (2014). SCOP2 prototype: A new approach to protein structure mining. Nucleic Acids Res.

[98] Leaver-Fay A, Tyka M, Lewis SM, Lange OF, Thompson J, Jacak R (2011). ROSETTA3: an object-oriented software suite for the simulation and design of macromolecules. Methods Enzymol.

[99] Abbasi E, Ghatee M, Shiri ME (2013). FRAN and RBF-PSO as two components of a hyper framework to recognize protein folds. Comput Biol Med.

[100] Kavousi K, Moshiri B, Sadeghi M, Araabi BN, Moosavi-Movahedi AA (2011). A protein fold classifier formed by fusing different modes of pseudo amino acid composition via PSSM. Comput Biol Chem.

[101] Giorgetti A, Raimondo D, Miele AE, Tramontano A (2005). Evaluating the usefulness of protein structure models for molecular replacement. Bioinformatics.

[102] Shi S, Pei J, Sadreyev RI, Kinch LN, Majumdar I, Tong J (2009). Analysis of CASP8 targets, predictions and assessment methods. Database (Oxford).

[103] Zhang J, Wang Q, Barz B, He Z, Kosztin I, Shang Y (2010). MUFOLD: A new solution for protein 3D structure prediction. Proteins.

[104] Kalman M, Ben-Tal N (2010). Quality assessment of protein model-structures using evolutionary conservation. Bioinformatics.

[105] Kim DE, Chivian D, Baker D (2004). Protein structure prediction and analysis using the Robetta server. Nucleic Acids Res.

[106] Gront D, Kulp DW, Vernon RM, Strauss CEM, Baker D (2011). Generalized fragment picking in Rosetta: design, protocols and applications. PLoS One.

[107] Kabsch W, Sander C (1983). Dictionary of protein secondary structure: pattern recognition of hydrogen-bonded and geometrical features. Biopolymers.

[108] Zemla A (2003). LGA: a method for finding 3D similarities in protein structures. Nucleic Acids Res.

[109] Siew N, Elofsson A, Rychlewski L, Fischer D (2000). MaxSub: an automated measure for the assessment of protein structure prediction quality. Bioinformatics.

