# Supplementary material for: Customised fragments libraries for protein structure prediction based on structural class annotations
Source: BMC Bioinformatics. 2015 Apr 29;16(1):136. doi: 10.1186/s12859-015-0576-2 (PMC4419399; doi:10.1186/s12859-015-0576-2)
Supplement: Additional file 1: Table S1. — It includes the detailed results for the 70 targets using three metrics: GDT_TS, GDT_HA and RMSD for the three experiments (Standard, CATH-based and SCOP-based). For each experiment two sets of data are provided; the best and the average of the best 10 scores of each metric. [file 12859_2015_576_MOESM1_ESM.docx]

**Additional file 1: Table S1. Detailed results of the 70 targets.**

For each target, three results were show: the standard predictions, CATH-based Predictions and SCOP-based Predictions; each assessed with three metrics. The first 15 targets are classified as FM, whereas the remaining are TBM. At least, 60,000 conformations were generated for each structure: 20,000 for the standard experiments, 20,000 for CATH and 20,000 for SCOP. For each experiment, at least six numbers (two rows) are presented: the first row is the first best value and the second one is the average of the best 10 values for each metric. Some targets comprise more than one experiment for CATH and SCOP, this is due to one of two reasons; either to study wrongly predicted targets by some class predictors or because of some targets’ secondary structure contents are in the overlapped region of two classes. The percentage of Helices and Sheets are as shown in the PDB and produced by the DSSP. Classes that are shown in bold were annotated by CATH/SCOP in the PDB. Classes that are shown in Italic are formally annotated, however, based on a domain which quite smaller than the whole chain.

|  | Target Code / PDB Code | Category  Percentage of H/E | Len | Standard Prediction | | | CATH-based Predictions | | | | SCOP-based predictions | | | |
| --- | --- | --- | --- | --- | --- | --- | --- | --- | --- | --- | --- | --- | --- | --- |
|  |  |  |  | Highest GDT_TS | Highest GDT_HA | Lowest RMSD | Highest GDT_TS | Highest GDT_HA | Lowest  RMSD | Class | Highest GDT_TS | Highest GDT_HA | Lowest  RMSD | Class |
| 1 | R0018/4EBG | CASP ROLL | 96 | 48.43 | 27.34 | 4.9 | 47.39 | 27.60 | 4.6 | Alpha Beta | 45.05 | 27.34 | 4.3 | Alpha + Beta |
|  | Top 10 | 33/28 |  | 42.00 | 24.97 | 5.3 | 43.77 | 24.84 | 5.0 |  | 42.39 | 24.97 | 5.2 |  |
| 2 | R0008/4EW7 | CASP ROLL | 113 | 46.23 | 26.10 | 4.8 | 53.54 | 31.19 | 3.8 | Alpha Beta | 52.21 | 33.18 | 4.5 | Alpha + Beta |
|  | Top 10 | 31/28 |  | 43.18 | 24.92 | 5.5 | 47.52 | 29.26 | 4.7 |  | 48.29 | 31.26 | 4.9 |  |
|  |  |  |  |  |  |  | 47.78 | 27.87 | 4.8 | Mainly Beta | 47.34 | 30.31 | 5.1 | All Beta |
|  |  |  |  |  |  |  | 42.94 | 26.26 | 5.8 |  | 41.43 | 26.43 | 6.1 |  |
| 3 | R0020/4HLB | CASP ROLL | 95 | 51.05 | 32.10 | 3.8 | 48.42 | 30.52 | 4.1 | Alpha Beta | 47.89 | 30.00 | 5.2 | Alpha + Beta |
|  | Top 10 | 28/24 |  | 48.78 | 30.44 | 5.0 | 45.26 | 28.60 | 5.3 |  | 45.23 | 28.60 | 5.4 |  |
| 4 | R0020/4IAJ | CASP ROLL | 76 | 65.46 | 48.35 | 3.0 | 75.00 | 53.61 | 2.4 | Alpha Beta | 72.03 | 51.64 | 2.6 | Alpha + Beta |
|  | Top 10 | 25/34 |  | 63.09 | 42.10 | 3.3 | 66.38 | 46.05 | 3.1 |  | 67.76 | 47.73 | 3.0 |  |
| 5 | R0034/4L3U | CASP ROLL | 123 | 60.36 | 43.29 | 4.2 | 67.27 | 47.56 | 3.4 | Mainly Alpha | 59.14 | 41.05 | 4.5 | All Alpha |
|  | Top 10 | 62/1 |  | 57.64 | 39.95 | 4.8 | 60.20 | 40.75 | 4.3 |  | 55.83 | 37.17 | 4.7 |  |
| 6 | R0039/4LQZ | CASP ROLL | 124 | 23.58 | 13.30 | 9.1 | 24.79 | 14.31 | 9.6 | Mainly Beta | 28.02 | 15.12 | 9.3 | All Beta |
|  | Top 10 | 6/43 |  | 22.60 | 12.27 | 9.8 | 23.30 | 12.88 | 10.0 |  | 23.74 | 13.16 | 9.8 |  |
| 7 | T0460/2K4N | CASP8 FM | 102 | 38.48 | 26.47 | 7.7 | 45.09 | 27.69 | 4.5 | Alpha Beta | 49.26 | 34.06 | 5.6 | Alpha + Beta |
|  | Top 10 | 28/27 |  | 35.68 | 24.77 | 8.6 | 42.54 | 26.71 | 6.0 |  | 40.88 | 27.57 | 7.1 |  |
|  |  |  |  |  |  |  | 44.60 | 28.18 | 5.5 | Mainly Alpha | 43.13 | 27.45 | 7.0 | All Alpha |
|  |  |  |  |  |  |  | 40.09 | 26.86 | 7.0 |  | 39.65 | 25.98 | 7.3 |  |
| 8 | T0482/2K4V | CASP8 FM | 125 | 38.80 | 26.00 | 8.8 | 37.20 | 25.80 | 10 | Alpha Beta | 34.60 | 23.00 | 8.7 | Alpha + Beta |
|  | Top 10 | 28/32 |  | 36.00 | 24.28 | 9.6 | 34.72 | 22.94 | 10.3 |  | 32.98 | 21.38 | 9.8 |  |
| 9 | T0531/2KJX | CASP9 FM | 65 | 39.61 | 25.76 | 7.0 | 49.23 | 29.61 | 6.1 | Alpha Beta | 43.46 | 28.46 | 6.7 | Alpha + Beta |
|  | Top 10 | 20/33 |  | 37.84 | 23.23 | 7.6 | 45.07 | 27.00 | 6.6 |  | 40.42 | 26.07 | 7.0 |  |
|  |  |  |  |  |  |  | 41.15 | 24.23 | 6.3 | Mainly Beta | 44.61 | 28.46 | 5.9 | All Beta |
|  |  |  |  |  |  |  | 39.65 | 23.34 | 6.8 |  | 41.65 | 25.57 | 6.6 |  |
|  |  |  |  |  |  |  | 45.38 | 30.38 | 5.9 | FSS | 48.07 | 29.23 | 5.8 | Small Proteins |
|  |  |  |  |  |  |  | 42.92 | 26.00 | 6.5 |  | 43.99 | 26.61 | 6.3 |  |
| 10 | T0553/2KY4 | CASP9 FM | 141 | 51.24 | 30.14 | 5.4 | 43.08 | 23.21 | 5.6 | Mainly Alpha | 45.56 | 25.88 | 5.0 | All Alpha |
|  | Top 10 | 59/1 |  | 43.08 | 27.26 | 6.1 | 39.36 | 21.87 | 6.1 |  | 40.31 | 23.97 | 6.0 |  |
| 11 | T0639/3NYM | CASP9 FM | 123 | 45.73 | 38.00 | 6.8 | 45.93 | 35.77 | 5.1 | Mainly Alpha | 45.52 | 37.80 | 6.2 | All Alpha |
|  | Top 10 | 59/3 |  | 44.04 | 35.26 | 7.6 | 43.61 | 34.02 | 6.8 |  | 43.73 | 35.32 | 7.5 |  |
| 12 | T0637/2X3O | CASP9 FM | 135 | 44.81 | 30.00 | 8.2 | 43.14 | 29.25 | 5.8 | Mainly Alpha | 50.74 | 31.48 | 8.5 | All Alpha |
|  | Top 10 | 78/0 |  | 41.27 | 27.11 | 8.9 | 40.38 | 26.96 | 8.9 |  | 44.24 | 27.51 | 9.6 |  |
| 13 | T0624/3NRL | CASP9 FM | 69 | 50.00 | 31.52 | 5.2 | 51.81 | 32.97 | 4.5 | Mainly Beta | 51.81 | 31.52 | 4.5 | All Beta |
|  | Top 10 | 0/46 |  | 45.18 | 28.94 | 5.7 | 47.86 | 30.79 | 5.3 |  | 46.84 | 29.96 | 5.0 |  |
| 14 | T0581/3NPD | CASP9 FM | 113 | 41.37 | 28.09 | 7.3 | 40.70 | 27.87 | 6.2 | Alpha Beta | 39.15 | 26.99 | 7.7 | Alpha + Beta |
|  | Top 10 | 43/28 |  | 37.34 | 24.82 | 8.3 | 39.09 | 26.19 | 7.5 |  | 37.78 | 24.97 | 8.1 |  |
|  |  |  |  |  |  |  | 42.03 | 28.09 | 7.8 | Mainly Alpha | 39.15 | 26.32 | 7.2 | All Alpha |
|  |  |  |  |  |  |  | 37.01 | 25.77 | 8.6 |  | 35.90 | 25.24 | 8.2 |  |
| 15 | T0855/2MQD | CASP11 FM | 119 | 37.39 | 22.26 | 6.4 | 43.48 | 26.89 | 5.2 | Alpha Beta | 42.43 | 25.84 | 5.4 | Alpha + Beta |
|  | Top 10 | 29/23 |  | 35.54 | 21.80 | 6.9 | 40.25 | 24.91 | 6.4 |  | 39.28 | 23.50 | 6.1 |  |
|  |  |  |  |  |  |  | 44.74 | 24.79 | 5.1 | Mainly Beta | 50.21 | 27.31 | 4.6 | All Beta |
|  |  |  |  |  |  |  | 39.70 | 23.67 | 6.0 |  | 38.44 | 23.25 | 5.8 |  |
| 16 | T0480/2K4X | CASP8 TBM | 55 | 49.09 | 34.09 | 8.3 | 43.18 | 29.54 | 9.6 | FSS | 45.45 | 28.63 | 9.2 | **Small Proteins** |
|  | Top 10 | 0/12 |  | 47.00 | 31.59 | 9.0 | 41.00 | 26.13 | 9.7 |  | 43.36 | 27.90 | 9.5 |  |
| 17 | T0468/2K5W | CASP8 TBM | 111 | 35.36 | 19.36 | 7.7 | 34.23 | 20.27 | 7.5 | Mainly Beta | 38.96 | 20.49 | 7.3 | **All Beta** |
|  | Top 10 | 9/33 |  | 31.41 | 18.15 | 8.9 | 31.19 | 18.60 | 8.2 |  | 34.12 | 19.03 | 8.0 |  |
| 18 | T0475/2K54 | CASP8 TBM | 123 | 38.08 | 22.56 | 6.1 | 40.04 | 28.04 | 6.5 | Alpha Beta | 42.07 | 29.47 | 5.1 | Alpha + Beta |
|  | Top 10 | 29/47 |  | 35.00 | 21.05 | 7.0 | 38.08 | 26.64 | 7.0 |  | 40.28 | 27.45 | 6.6 |  |
| 19 | T0472/2K49 | CASP8 TBM | 118 | 41.73 | 25.63 | 6.9 | 45.33 | 28.39 | 6.5 | Alpha Beta | 41.73 | 24.15 | 7.7 | **Alpha + Beta** |
|  | Top 10 | 26/32 |  | 38.24 | 24.47 | 8.1 | 41.80 | 26.05 | 7.8 |  | 38.81 | 23.24 | 8.1 |  |
| 20 | T0451/3DMC | CASP8 TBM | 133 | 40.97 | 24.06 | 5.4 | 38.91 | 25.75 | 6.0 | Alpha Beta | 36.46 | 23.87 | 6.5 | Alpha + Beta |
|  | Top 10 | 29/46 |  | 37.63 | 23.32 | 6.5 | 36.53 | 23.57 | 6.8 |  | 33.92 | 21.71 | 7.0 |  |
| 21 | T0507/3DO8 | CASP8 TBM | 142 | 49.39 | 29.43 | 5.2 | 50.00 | 31.65 | 5.0 | **Alpha Beta** | 53.22 | 33.66 | 5.1 | Alpha/Beta |
|  | Top 10 | 34/17 |  | 40.04 | 25.62 | 7.0 | 43.83 | 27.74 | 6.7 |  | 44.39 | 28.18 | 6.0 |  |
|  |  |  |  |  |  |  |  |  |  |  | 37.90 | 23.79 | 6.2 | Alpha + Beta |
|  |  |  |  |  |  |  |  |  |  |  | 35.02 | 22.62 | 7.1 |  |
| 22 | T0459/3DF8 | CASP8 TBM | 106 | 57.31 | 39.38 | 5.3 | 69.57 | 53.77 | 5.1 | **Mainly Alpha** | 70.04 | 53.06 | 4.8 | All Alpha |
|  | Top 10 | 48/18 |  | 52.28 | 35.00 | 5.6 | 65.91 | 49.31 | 5.3 |  | 66.15 | 50.68 | 5.1 |  |
|  |  |  |  |  |  |  | 73.82 | 56.60 | 4.9 | Alpha Beta | 57.07 | 41.03 | 5.2 | Alpha + Beta |
|  |  |  |  |  |  |  | 67.21 | 50.70 | 5.2 |  | 55.09 | 37.99 | 5.7 |  |
| 23 | T0458/3DEX | CASP8 TBM | 79 | 63.92 | 42.72 | 3.0 | 69.30 | 46.51 | 2.7 | **Alpha Beta** | 77.84 | 55.69 | 2.4 | Alpha + Beta |
|  | Top 10 | 25/25 |  | 61.74 | 40.12 | 3.3 | 61.58 | 39.33 | 3.3 |  | 68.25 | 47.02 | 2.6 |  |
| 24 | T0466/3DCX | CASP8 TBM | 116 | 40.08 | 25.21 | 6.5 | 34.26 | 21.12 | 6.1 | Mainly Beta | 40.30 | 23.06 | 6.0 | **All Beta** |
|  | Top 10 | 24/40 |  | 35.38 | 21.65 | 6.8 | 32.90 | 18.90 | 6.6 |  | 34.05 | 19.91 | 6.0 |  |
|  |  |  |  |  |  |  | 36.85 | 20.47 | 5.7 | Alpha Beta | 45.90 | 22.84 | 4.8 | Alpha + Beta |
|  |  |  |  |  |  |  | 34.65 | 19.39 | 6.4 |  | 35.38 | 20.28 | 6.1 |  |
| 25 | T0499/2KDM | CASP8 TBM | 56 | 79.01 | 57.58 | 1.7 | 75.44 | 52.67 | 1.9 | **Alpha Beta** | 82.14 | 59.37 | 1.4 | Alpha + Beta |
|  | Top 10 | 25/46 |  | 73.48 | 51.20 | 2.0 | 68.52 | 45.35 | 2.3 |  | 78.39 | 55.00 | 1.7 |  |
| 26 | T0411/3D1P | CASP8 TBM | 120 | 41.45 | 28.33 | 6.6 | 48.75 | 30.62 | 5.6 | **Alpha Beta** | 57.70 | 36.85 | 5.0 | Alpha/Beta |
|  | Top 10 | 34/12 |  | 38.37 | 26.12 | 7.5 | 43.27 | 26.93 | 6.6 |  | 52.93 | 34.54 | 6.0 |  |
| 27 | T0498/2KDL | CASP8 TBM | 56 | 41.51 | 26.78 | 7.1 | 44.64 | 29.46 | 5.9 | **Mainly Alpha** | 46.42 | 30.80 | 6.3 | All Alpha |
|  | Top 10 | 62/0 |  | 38.70 | 25.04 | 7.2 | 40.80 | 27.05 | 6.8 |  | 45.05 | 29.15 | 6.4 |  |
|  |  |  |  |  |  |  | 39.73 | 24.10 | 6.7 | Alpha Beta | 41.51 | 24.10 | 6.4 | Alpha + Beta |
|  |  |  |  |  |  |  | 35.89 | 22.00 | 7.4 |  | 39.01 | 23.30 | 6.7 |  |
| 28 | T0415/3D6W | CASP8 TBM | 109 | 75.68 | 51.83 | 2.0 | 80.50 | 65.8 | 2.3 | Alpha Beta | 72.2 | 50.00 | 2.1 | Alpha + Beta |
|  | Top 10 | 18/45 |  | 66.99 | 43.83 | 2.7 | 70.66 | 48.64 | 2.6 |  | 68.39 | 45.82 | 2.5 |  |
|  |  |  |  |  |  |  | 72.24 | 49.31 | 2.4 | ***Mainly Beta*** | 62.38 | 41.51 | 2.7 | All Beta |
|  |  |  |  |  |  |  | 62.70 | 38.83 | 2.8 |  | 59.72 | 36.16 | 3.1 |  |
| 29 | T0414/3D0J | CASP8 TBM | 137 | 33.21 | 19.89 | 8.5 | 36.49 | 23.90 | 8.0 | **Mainly Beta** | 35.21 | 21.35 | 7.7 | All Beta |
|  | Top 10 | 14/45 |  | 30.25 | 17.18 | 9.5 | 32.35 | 18.61 | 9.1 |  | 32.06 | 18.83 | 8.9 |  |
| 30 | T0488/2VWR | CASP8 TBM | 95 | 50.78 | 32.89 | 6.6 | 60.79 | 41.57 | 3.6 | **Mainly Beta** | 78.42 | 59.21 | 2.2 | All Beta |
|  | Top 10 | 15/37 |  | 44.15 | 28.97 | 7.2 | 57.18 | 38.52 | 4.8 |  | 74.21 | 53.71 | 2.9 |  |
|  |  |  |  |  |  |  | 58.42 | 38.94 | 3.9 | Alpha Beta | 50.26 | 33.42 | 4.4 | Alpha + Beta |
|  |  |  |  |  |  |  | 53.81 | 34.60 | 4.8 |  | 43.42 | 28.94 | 6.6 |  |
| 31 | T0409/3D0F | CASP8 TBM | 105 | 47.61 | 35.00 | 8.0 | 48.33 | 34.04 | 7.8 | **Mainly Beta** | 52.61 | 40.23 | 8.4 | All Beta |
|  | Top 10 | 16/30 |  | 42.49 | 29.78 | 9.4 | 43.59 | 30.04 | 9.1 |  | 45.47 | 31.11 | 9.0 |  |
|  |  |  |  |  |  |  | 46.90 | 33.57 | 8.5 | Alpha Beta | 40.23 | 26.66 | 8.4 | Alpha + Beta |
|  |  |  |  |  |  |  | 41.66 | 28.59 | 9.4 |  | 38.00 | 25.47 | 9.2 |  |
| 32 | T0455/3DDV | CASP8 TBM | 139 | 35.43 | 23.38 | 7.5 | 61.15 | 38.66 | 3.7 | **Alpha Beta** | 52.69 | 33.27 | 6.8 | **Alpha + Beta** |
|  | Top 10 | 24/44 |  | 32.75 | 20.91 | 8.5 | 48.92 | 33.61 | 6.0 |  | 41.45 | 32.14 | 7.4 |  |
| 33 | T0453/3DED | CASP8 TBM | 86 | 54.65 | 33.43 | 4.0 | 56.10 | 34.59 | 4.0 | **Alpha Beta** | 62.20 | 43.60 | 3.3 | **Alpha + Beta** |
|  | Top 10 | 17/27 |  | 50.40 | 30.20 | 4.3 | 52.44 | 31.80 | 4.2 |  | 60.81 | 40.90 | 3.6 |  |
| 34 | T0432/3DAI | CASP8 TBM | 128 | 50.97 | 31.44 | 4.0 | 69.72 | 48.24 | 2.7 | Mainly Alpha | 53.32 | 33.20 | 3.5 | All Alpha |
|  | Top 10 | 73/0 |  | 47.83 | 27.63 | 5.0 | 62.26 | 40.50 | 3.3 |  | 49.98 | 30.62 | 4.2 |  |
| 35 | T0498/3KLW | CASP8 TBM | 98 | 41.07 | 29.08 | 7.2 | 58.92 | 40.81 | 4.3 | Mainly Beta | 60.71 | 42.34 | 4.3 | All Beta |
|  | Top 10 | 6/65 |  | 36.55 | 22.09 | 8.9 | 53.62 | 35.22 | 5.2 |  | 55.96 | 37.88 | 5.4 |  |
|  |  |  |  |  |  |  | 50.00 | 32.90 | 6.3 | Alpha Beta | 50.00 | 32.39 | 5.1 | Alpha + Beta |
|  |  |  |  |  |  |  | 45.35 | 28.6226 | 6.8 |  | 46.65 | 30.15 | 6.7 |  |
| 36 | T0502/3DM3 | CASP8 TBM | 98 | 77.04 | 55.10 | 2.0 | 61.48 | 39.03 | 3.1 | Mainly Beta | 51.78 | 36.48 | 4.4 | All Beta |
|  | Top 10 | 7/59 |  | 70.05 | 49.36 | 2.9 | 54.20 | 34.54 | 4.7 |  | 48.36 | 31.68 | 5.9 |  |
| 37 | T0619/3NRW | CASP9 TBM | 104 | 67.30 | 50.00 | 2.8 | 76.20 | 53.36 | 2.0 | Mainly Alpha | 79.08 | 58.17 | 1.8 | All Alpha |
|  | Top 10 | 59/0 |  | 63.60 | 42.54 | 3.1 | 69.00 | 46.12 | 2.6 |  | 69.54 | 48.07 | 2.5 |  |
| 38 | T0539/2L0B | CASP9 TBM | 68 | 43.75 | 25.73 | 5.7 | 47.42 | 30.51 | 6.1 | FSS | 56.61 | 41.17 | 5.0 | Small Proteins |
|  | Top 10 | 14/19 |  | 36.32 | 23.52 | 6.6 | 42.46 | 28.19 | 6.7 |  | 52.16 | 37.46 | 5.7 |  |
|  |  |  |  |  |  |  | 40.38 | 29.39 | 8.1 | Alpha Beta | 36.81 | 26.39 | 8.1 | Alpha + Beta |
|  |  |  |  |  |  |  | 39.14 | 27.63 | 8.7 |  | 33.68 | 22.17 | 9.1 |  |
| 39 | T0630/2KYT | CASP9 TBM | 125 | 29.20 | 16.80 | 8.7 | 32.80 | 21.00 | 7.6 | Alpha Beta | 35.20 | 17.80 | 6.9 | Alpha + Beta |
|  | Top 10 | 28/24 |  | 26.64 | 15.68 | 9.7 | 29.78 | 17.50 | 8.6 |  | 28.84 | 16.46 | 9.1 |  |
|  |  |  |  |  |  |  |  |  |  |  | 31.60 | 18.80 | 7.5 | Alpha/Beta |
|  |  |  |  |  |  |  |  |  |  |  | 28.80 | 15.68 | 8.6 |  |
| 40 | T0619/2L0D | CASP9 TBM | 114 | 33.99 | 22.14 | 8.0 | 37.28 | 22.36 | 7.1 | Mainly Beta | 36.40 | 23.68 | 7.4 | All Beta |
|  | Top 10 | 2/54 |  | 32.14 | 20.41 | 9.0 | 35.43 | 21.11 | 7.6 |  | 34.23 | 21.88 | 8.2 |  |
| 41 | T0522/3NRD | CASP9 TBM | 134 | 36.56 | 24.06 | 8.1 | 44.03 | 26.30 | 6.6 | Alpha Beta | 39.55 | 25.18 | 7.1 | Alpha + Beta |
|  | Top 10 | 35/22 |  | 34.83 | 23.30 | 8.6 | 39.32 | 24.92 | 7.9 |  | 37.55 | 23.75 | 8.0 |  |
| 42 | T0540/3MX7 | CASP9 TBM | 90 | 47.50 | 30.83 | 4.5 | 43.33 | 30.83 | 5.1 | Mainly Beta | 45.55 | 32.77 | 5.7 | All Beta |
|  | Top 10 | 0/65 |  | 44.80 | 29.49 | 4.8 | 41.58 | 29.19 | 6.0 |  | 42.30 | 29.02 | 6.0 |  |
|  |  |  |  |  |  |  | 41.94 | 27.22 | 5.3 | Alpha Beta | 43.88 | 29.44 | 5.0 | Alpha + Beta |
|  |  |  |  |  |  |  | 38.97 | 26.13 | 5.7 |  | 38.91 | 26.41 | 5.9 |  |
| 43 | T0523/3MQO | CASP9 TBM | 113 | 45.57 | 26.10 | 5.8 | 49.77 | 33.18 | 4.5 | Alpha Beta | 49.77 | 27.87 | 5.3 | Alpha + Beta |
|  | Top 10 | 32/31 |  | 37.67 | 23.51 | 6.4 | 45.53 | 27.27 | 5.3 |  | 43.03 | 24.62 | 5.7 |  |
| 44 | T0538/2L09 | CASP9 TBM | 53 | 98.11 | 84.90 | 0.7 | 97.64 | 85.84 | 0.6 | Mainly Alpha | 99.52 | 91.03 | 0.6 | All Alpha |
|  | Top 10 | 62/6 |  | 96.50 | 81.88 | 0.8 | 96.74 | 82.35 | 0.8 |  | 98.44 | 87.97 | 0.7 |  |
| 45 | T0574/3NRF | CASP9 TBM | 102 | 39.46 | 22.79 | 6.6 | 46.56 | 27.94 | 6.1 | Mainly Beta | 40.19 | 21.56 | 6.8 | All Beta |
|  | Top 10 | 5/57 |  | 36.64 | 21.10 | 7.7 | 39.65 | 22.72 | 6.8 |  | 36.76 | 20.90 | 7.9 |  |
| 46 | T0622/3NKL | CASP9 TBM | 121 | 53.09 | 31.40 | 4.5 | 52.06 | 34.50 | 4.2 | Alpha Beta | 57.85 | 36.77 | 4.4 | Alpha/Beta |
|  | Top 10 | 34/20 |  | 48.69 | 29.27 | 5.0 | 49.09 | 30.18 | 4.8 |  | 50.22 | 30.82 | 4.9 |  |
| 47 | T0580/3NBM | CASP9 TBM | 104 | 52.16 | 32.69 | 3.5 | 65.38 | 41.34 | 2.7 | Alpha Beta | 58.65 | 34.85 | 2.8 | Alpha/Beta |
|  | Top 10 | 47/20 |  | 49.20 | 30.76 | 3.9 | 58.74 | 36.53 | 3.2 |  | 54.03 | 33.24 | 3.4 |  |
| 48 | T0643/3NZL | CASP9 TBM | 73 | 65.06 | 42.46 | 2.7 | 71.57 | 52.37 | 2.8 | Mainly Alpha | 67.80 | 44.52 | 2.9 | All Alpha |
|  | Top 10 | 59/0 |  | 59.55 | 40.44 | 3.4 | 64.21 | 43.59 | 3.0 |  | 60.37 | 38.73 | 3.2 |  |
| 49 | T0711/2M7T | CASP10 TBM | 33 | 66.66 | 51.51 | 2.9 | 59.84 | 44.69 | 3.3 | FSS | 71.21 | 59.09 | 2.4 | Small Proteins |
|  | Top 10 | 9/18 |  | 57.04 | 38.78 | 3.3 | 55.53 | 37.80 | 3.5 |  | 68.73 | 52.87 | 2.8 |  |
| 50 | T0665/2LR8 | CASP10 TBM | 70 | 67.87 | 58.21 | 4.7 | 68.57 | 57.50 | 4.7 | Mainly Alpha | 66.07 | 51.42 | 4.9 | All Alpha |
|  | Top 10 | 57/0 |  | 66.57 | 54.14 | 5.3 | 65.85 | 53.89 | 5.3 |  | 62.35 | 47.89 | 5.2 |  |
| 51 | T0716/2LY9 | CASP10 TBM | 74 | 66.55 | 51.68 | 4.4 | 67.56 | 52.36 | 5.6 | Mainly Alpha | 67.23 | 53.37 | 6.0 | All Alpha |
|  | Top 10 | 51/0 |  | 64.45 | 47.97 | 5.8 | 64.66 | 49.29 | 6.4 |  | 64.59 | 49.56 | 6.4 |  |
| 52 | T0675/2LV2 | CASP10 TBM | 85 | 33.52 | 21.76 | 9.5 | 34.70 | 23.23 | 9.3 | Alpha Beta | 34.11 | 22.64 | 9.8 | Alpha + Beta |
|  | Top 10 | 21/9 |  | 29.67 | 21.11 | 10.3 | 33.52 | 22.20 | 10.0 |  | 33.20 | 22.00 | 10.4 |  |
|  |  |  |  |  |  |  | 30.58 | 17.94 | 9.0 | FSS | 34.41 | 22.35 | 8.6 | Small Proteins |
|  |  |  |  |  |  |  | 28.44 | 16.73 | 10.3 |  | 30.73 | 21.70 | 9.9 |  |
| 53 | T0731/2LZ1 | CASP10 TBM | 90 | 48.88 | 38.05 | 7.8 | 53.05 | 41.66 | 7.6 | Mainly Alpha | 51.94 | 39.44 | 7.6 | All Alpha |
|  | Top 10 | 42/0 |  | 46.27 | 33.77 | 8.4 | 49.33 | 36.88 | 8.3 |  | 47.52 | 34.47 | 8.8 |  |
| 54 | T0754/2LV9 | CASP10 TBM | 80 | 44.06 | 27.81 | 6.3 | 41.56 | 28.12 | 7.1 | Alpha Beta | 35.62 | 26.25 | 7.3 | Alpha + Beta |
|  | Top 10 | 14/10 |  | 39.90 | 26.71 | 7.1 | 39.50 | 27.00 | 7.8 |  | 34.87 | 23.90 | 7.8 |  |
|  |  |  |  |  |  |  | 34.68 | 21.56 | 7.2 | FSS | 43.75 | 33.43 | 7.5 | Small Proteins |
|  | Top 10 |  |  |  |  |  | 32.56 | 20.78 | 8.1 |  | 37.59 | 27.37 | 8.1 |  |
| 55 | T0727/2LTM | CASP10 TBM | 107 | 49.76 | 34.11 | 4.7 | 52.33 | 35.51 | 5.8 | Alpha Beta | 50.46 | 36.21 | 5.9 | Alpha + Beta |
|  | Top 10 | 28/22 |  | 47.47 | 32.35 | 6.2 | 48.69 | 34.97 | 6.7 |  | 47.80 | 33.85 | 6.7 |  |
| 56 | T0669/2LTL | CASP10 TBM | 119 | 42.85 | 29.41 | 7.1 | 49.79 | 33.61 | 7.4 | Alpha Beta | 45.79 | 32.14 | 7.2 | Alpha + Beta |
|  | Top 10 | 27/21 |  | 40.75 | 26.02 | 7.8 | 41.72 | 27.05 | 7.9 |  | 44.15 | 30.84 | 7.9 |  |
| 57 | T0753/4GOQ | CASP10 TBM | 108 | 48.38 | 28.00 | 5.1 | 49.30 | 31.71 | 4.3 | Alpha Beta | 57.63 | 35.88 | 4.4 | Alpha + Beta |
|  | Top 10 | 25/30 |  | 41.80 | 25.02 | 6.0 | 45.71 | 27.19 | 5.2 |  | 46.52 | 28.77 | 5.4 |  |
| 58 | T0680/4FM3 | CASP10 TBM | 94 | 90.95 | 71.80 | 1.1 | 94.14 | 81.11 | 0.8 | Mainly Alpha | 91.75 | 75.53 | 0.9 | All Alpha |
|  | Top 10 | 80/0 |  | 83.24 | 68.13 | 1.7 | 93.43 | 78.77 | 0.9 |  | 90.00 | 72.73 | 1.1 |  |
| 59 | T0747/4G5A | CASP10 TBM | 98 | 35.96 | 22.19 | 9.3 | 36.99 | 25.51 | 8.9 | Mainly Beta | 34.18 | 20.91 | 8.3 | All Beta |
|  | Top 10 | 4/53 |  | 32.49 | 20.35 | 9.7 | 34.20 | 20.58 | 9.7 |  | 33.01 | 20.21 | 9.1 |  |
| 60 | T0723/4FLB | CASP10 TBM | 131 | 62.78 | 39.69 | 3.4 | 59.54 | 36.45 | 3.0 | Mainly Alpha | 58.20 | 37.21 | 3.3 | All Alpha |
|  | Top 10 | 68/0 |  | 52.51 | 31.98 | 3.9 | 56.03 | 34.31 | 3.5 |  | 53.85 | 32.99 | 3.7 |  |
| 61 | T0743/4HYZ | CASP10 TBM | 113 | 49.55 | 31.63 | 5.1 | 53.98 | 31.63 | 4.7 | Alpha Beta | 50.44 | 29.42 | 5.0 | Alpha + Beta |
|  | Top 10 | 32/38 |  | 44.31 | 27.89 | 5.6 | 46.57 | 28.89 | 5.2 |  | 46.17 | 27.80 | 5.5 |  |
| 62 | T0769/2MQ8 | CASP11 TBM | 112 | 66.51 | 45.75 | 4.5 | 59.37 | 37.94 | 4.7 | Alpha Beta | 69.8 | 48.21 | 4.8 | Alpha + Beta |
|  | Top 10 | 37/31 |  | 62.58 | 41.40 | 5.5 | 58.77 | 37.12 | 5.5 |  | 65.37 | 44.21 | 5.5 |  |
| 63 | T0857/2MQC | CASP11 TBM | 105 | 30.71 | 17.61 | 8.7 | 29.52 | 17.38 | 9.5 | Mainly Beta | 30.47 | 20.71 | 9.3 | All Beta |
|  | Top 10 | 0/45 |  | 28.04 | 16.73 | 9.8 | 27.52 | 16.33 | 9.9 |  | 28.881 | 17.5954 | 10.1 |  |
| 64 | T0782/4QRL | CASP11 TBM | 110 | 50 | 32.27 | 4.7 | 50.90 | 31.81 | 4.9 | Mainly Beta | 45 | 27.50 | 5.4 | All Beta |
|  | Top 10 | 9/57 |  | 43.65 | 28.31 | 5.4 | 45.38 | 27.56 | 5.8 |  | 40.79 | 26.20 | 5.9 |  |
| 65 | T0833/4R03 | CASP11 TBM | 108 | 59.02 | 39.35 | 3.7 | 58.79 | 39.12 | 3.7 | Mainly Beta | 57.87 | 37.96 | 3.5 | All Beta |
|  | Top 10 | 7/70 |  | 53.93 | 35.85 | 4.1 | 54.62 | 35.64 | 4.2 |  | 54.65 | 35.02 | 4.2 |  |
| 66 | T0784/4QEY | CASP11 TBM | 125 | 27.8 | 16.4 | 9.5 | 27.4 | 15.8 | 10.1 | Mainly Beta | 23.4 | 14 | 9.5 | All Beta |
|  | Top 10 | 2/58 |  | 25.12 | 14.56 | 10.2 | 24.26 | 13.98 | 10.5 |  | 22.72 | 13.4 | 10.5 |  |
| 67 | T0766/4Q53 | CASP11 TBM | 108 | 43.98 | 30.78 | 4.8 | 47.68 | 31.48 | 4.8 | Mainly Beta | 50.23 | 27.08 | 4.4 | All Beta |
|  | Top 10 | 24/53 |  | 41.38 | 28.61 | 5.6 | 41.29 | 25.99 | 5.3 |  | 42.12 | 24.07 | 4.9 |  |
|  |  |  |  |  |  |  | 52.54 | 33.56 | 4.0 | Alpha Beta | 45.37 | 30.55 | 5.0 | Alpha + Beta |
|  | Top 10 |  |  |  |  |  | 45.50 | 29.81 | 5.2 |  | 43.86 | 27.87 | 5.5 |  |
| 68 | T0759/4Q28 | CASP11 TBM | 110 | 47.04 | 31.59 | 5.9 | 47.95 | 33.63 | 7.3 | Alpha Beta | 44.77 | 33.40 | 8.2 | Alpha + Beta |
|  | Top 10 | 33/30 |  | 42.40 | 30.11 | 7.2 | 43.97 | 32.54 | 8.0 |  | 43.43 | 31.56 | 9.0 |  |
| 69 | T0765/4PWU | CASP11 TBM | 76 | 76.64 | 55.59 | 2.4 | 75.98 | 55.26 | 2.3 | Alpha Beta | 67.10 | 50.98 | 2.7 | Alpha + Beta |
|  | Top 10 | 24/25 |  | 65.16 | 46.15 | 3.0 | 63.61 | 44.04 | 2.9 |  | 63.71 | 44.90 | 3.2 |  |
|  |  |  |  |  |  |  | 71.05 | 50.32 | 2.6 | Mainly Beta | 70.72 | 47.69 | 2.9 | All Beta |
|  |  |  |  |  |  |  | 65.50 | 47.17 | 3 |  | 62.10 | 43.74 | 3.2 |  |
| 70 | T0778/4CGW | CASP11 TBM | 115 | 74.78 | 60.21 | 5.8 | 88.04 | 73.26 | 1.4 | Mainly Alpha | 83.04 | 66.95 | 2.9 | All Alpha |
|  | Top 10 | 79/0 |  | 63.60 | 52.26 | 6.1 | 77.89 | 61.32 | 3.6 |  | 77.10 | 60.41 | 3.7 |  |
